# Opinion, knowledge, and clinical experience with functional neurological disorders among Italian neurologists: results from an online survey

**DOI:** 10.1007/s00415-021-10840-y

**Published:** 2021-10-19

**Authors:** Michele Tinazzi, Mirta Fiorio, Alfredo Berardelli, Bruno Bonetti, Domenico Marco Bonifati, Alessandro Burlina, Annachiara Cagnin, Francesca Calabria, Maurizio Corbetta, Pietro Cortelli, Bruno Giometto, Silvia Vittoria Guidoni, Leonardo Lopiano, Gianluigi Mancardi, Fabio Marchioretto, Maria Pellegrini, Francesco Teatini, Gioacchino Tedeschi, Lucia Tesolin, Emanuele Turinese, Mario Zappia, Angela Marotta

**Affiliations:** 1 Department of Neurosciences, Biomedicine and Movement Sciences, University of Verona, Verona, Italy; 2 Department of Human Neurosciences, Sapienza University of Rome, Rome, Italy; 3 Neurology Unit, Azienda Ospedaliera Universitaria Integrata Verona, Verona, Italy; 4 Neurology Unit, Cà Foncello Hospital, Treviso, Italy; 5 Neurology Unit, St Bassiano Hospital, Bassano del Grappa, Italy; 6 Department of Neuroscience, University of Padua, Padua, Italy; 7 Padova Neuroscience Center, University of Padua, Padua, Italy; 8 Venetian Institute of Molecular Medicine, VIMM Fondazione Biomedica Padova, Padua, Italy; 9 IRCCS, Istituto di Scienze Neurologiche di Bologna, Bologna, Italy; 10 DIBINEM, Alma Mater Studiorum, Università di Bologna, Bologna, Italy; 11 Department of Neurology, Santa Chiara Hospital, Trento, Italy; 12 Department of Neurosciences Rita Levi Montalcini, University of Torino, Turin, Italy; 13 Department of Neuroscience, Rehabilitation, Ophthalmology, Genetics, Maternal and Child, University of Genova, Genoa, Italy; 14 IRCCS ICS Maugeri, Pavia, Italy; 15 Neurology Unit, IRCCS Sacro Cuore Don Calabria Hospital, Negrar di Valpolicella, Verona Italy; 16 Functional Movement Disorders Outpatient Clinic, Clinical Neurology and Stroke Unit Department, Central Country Hospital, Bolzano, Italy; 17 Department of Advanced Medical and Surgical Sciences, University of Campania, Naples, Italy; 18 Department of Medical and Surgical Sciences and Advanced Technologies “G.F. Ingrassia”, Neuroscience Section, University of Catania, Catania, Italy; 19 IRCSS Neuromed, Pozzilli, Isernia, Italy; 20 AOU Città della Salute e della Scienza, Torino, Italy

**Keywords:** Functional neurological disorders, Conversion disorders, Neurological practice, Education, Survey

## Abstract

**Background:**

Functional neurological disorders (FND) are disabling medical conditions commonly seen in neurological practice. Neurologists play an essential role in managing FND, from establishing a diagnosis to coordination of multidisciplinary team-based treatment for patients. With this study, we investigated the knowledge and the clinical experience of Italian neurologists in managing patients with FND.

**Methods:**

Members of the Italian Society of Neurology were invited via e-mail to participate in this ad hoc online survey; 492 questionnaires were returned completed.

**Results:**

The term “Functional neurological disorders” in reference to FND was used more frequently than other psychological (e.g., psychogenic or conversion), or descriptive terms (e.g., non-organic or stress-related). When speaking with patients, the respondents stated that they preferred explaining symptoms based on abnormal functioning of the nervous system than discussing mental illness and that they would refer their patient to a psychologist rather than to a psychiatrist. Few considered that physiotherapy and psychiatric interventions are useful approaches to treating FND. Some believed that patients simulate their symptoms.

**Conclusions:**

Overall, the responses suggest that knowledge about scientific advances in FND is somewhat sparse. A psychiatric-centered view of FND opens the way to an approach in which neurobiological and psychological aspects constitute essential factors of the condition. In this context, professional education could improve understanding of FND and optimize patient management.

**Supplementary Information:**

The online version contains supplementary material available at 10.1007/s00415-021-10840-y.

## Introduction

Functional neurological disorders (FND) are disabling neurological conditions characterized by clinical signs that are incongruent with known neurological disease [1–3]. The etiology of FND has been long linked to psychological factors [4]. Research in the last decades has challenged this assumption, however, by demonstrating that the disease can be more consistently explained within a biopsychosocial framework in which neurobiological, psychological, and social factors are crucially involved in the etiology of the disease [5–7]. Recent findings have suggested new diagnostic approaches and treatment [8] in which diagnosis is based on cardinal clinical signs, such as inconsistency (remissions or exacerbations over time) with susceptibility to distraction (e.g., variation in tremor frequency and amplitude), and incongruity (discordance with other known neurological disorders) [3]. A multidisciplinary approach in which care is provided by a specialist team (e.g., neurologist, psychiatrist, psychotherapist, physiotherapist) is recommended [8, 9], with a growing body of evidence suggesting its efficacy for managing FND [10–14].

The neurologist plays a substantial role in diagnosis and in explaining the mechanisms of symptoms to the patient, suggesting appropriate treatment and follow-up, and coordinating a multidisciplinary approach [15]. Common in neurological practice [16, 17], FND are often found difficult to manage [18–20]. Previous studies suggested that absent or incomplete up-to-date knowledge of the disease might explain, at least in part, the difficulties in dealing with FND [20–25]. In this regard, several groups in diverse countries have surveyed neurologists for their opinion and clinical experience with FND to identify potential educational needs and implement novel strategies for developing an effective approach to the condition [20, 23–25]. Among these, LaFaver and colleagues have recently published the results of a survey that revealed a gap in the education of neurologists about the diagnosis of functional movement disorders based on positive clinical signs [24]. The survey involved only neurologists with expertise in movement disorders, leaving unexplored the opinion of neurologists from other specialties who also may encounter patients with FND. Moreover, the survey was administered to neurologists from different countries (e.g., the United States, Europe, Canada) and the data from Europe and Canada were pooled together, leaving open the question about potential differences in health care systems, training-related issues, cultural and social factors specific to the respondents’ geographical areas. Ad hoc investigations are needed to develop educational interventions based on local needs.

To the best of our knowledge, no study to date has surveyed Italian neurologists about their experience with FND. To fill this gap, we conducted an online survey of Italian neurologists. We invited neurologists in different specialties (e.g., movement disorders, epilepsy, cerebrovascular disease); this enabled us to investigate the opinions and the clinical experiences of a large cohort of neurologists who encounter a variety of FND phenotypes. The study is part of a larger project involving health professionals who encounter patients with FND in their clinical practice (e.g., neurologists, general practitioners, psychologists, psychiatrists, physiotherapists). In our previous study, we surveyed general practitioners about diagnosis, treatment, and management of FND patients [26]. The survey revealed the persistence among general practitioners of old myths about FND [26, 27], with a psychological view often prevailing over a multidisciplinary approach to patients with FND. For the present study, members of the Italian Society of Neurology (SIN) were invited to participate in a survey on how they manage this complex disorder.

## Methods

### Questionnaire

The questionnaire was based on the one used in our previous study that investigated the knowledge, opinions, and clinical experience of general practitioners in Italy [26]. Several questions were changed to better fit the professional interests of neurologists. New questions were added to explore how neurologists explain the symptoms to their patients and how they felt about their patients possibly feigning FND. The questions were created based on a literature review [19, 20, 23–25]. The questionnaire was reviewed by neurologists and modified according to their feedback. The final version consisted of 14 questions, as described below.

Five questions concerned demographics (age, sex, geographical area of residence) and professional characteristics (years of post-specialization and practice setting).

The other nine questions investigated opinion, knowledge and clinical experience with FND. Specifically, the first question investigated experience with FND (i.e., number of patients per week seen for neurological symptoms not explained by an organic cause). The second was a multiple-choice question about the terms they used to name FND. The third question focused on malingering and asked respondents to estimate on a scale from “not at all” to “very high” the probability that a patient deliberately feigned symptoms. The fourth was a single-choice question investigating how the respondent explained the disorder to patients. The fifth question regarded predictors of diagnosis. Respondents rated as “not at all”, “only a little”, “to some extent”, “a lot”, “very much” or “I don’t know” the extent to which they felt that each of eight items were predictive for a diagnosis of FND (e.g., reduction of symptoms with distractive maneuvers, changes in manifestations over time). The sixth question explored opinions about the appropriateness of specialist consultation and treatment and asked respondents to rate the degree of adequacy (from “not at all” to “very much” and “I don’t know”) of four items related to specialist consultations and five items describing different types of treatment for FND. The seventh question investigated the management strategies the respondents used to deal with their patients. The respondents stated the extent to which they agree (i.e., “totally disagree”, “disagree”, “uncertain,” “agree,” “totally agree”) with six items describing interventions in FND. The eighth question concerned their personal satisfaction with managing FND rated on a 11-point scale from 0 (no satisfaction) to 10 (high satisfaction). The ninth was a multiple-choice question on the neurologist’s role in FND diagnosis and treatment. All the survey questions are reported in the Supplementary Information.

### Procedure

Data were collected on an online platform (sin.neuro) for 8 weeks (4 June 2020–29 July 2020). Neurologists among the 2974 SIN members were invited to participate in the survey via an e-mail from the SIN. The e-mail explained that the aim of study was to investigate opinions, knowledge, and clinical experience with non-organic neurological disorders. The term non-organic was used to avoid connotations with presumed “functional” or “psychological” mechanisms underlying the disease. An example of what we meant by a non-organic disorder (e.g., neurological symptoms, like tremor, which may disappear with distraction) was also given to exclude potential bias due to misleading terminology. A survey link embedded in the e-mail and allowed direct access to the questionnaire. The first page of the questionnaire was a consent form, together with information about personal data handling (anonymity), and the time needed to complete the survey about 5 min. Respondents had to give their informed consent before they could start completing the questionnaire. Four e-mail reminders were sent by the SIN at 1, 2, 4, 6, and 7 weeks after the initial mailing. Response to all survey items was mandatory. If an item was skipped, a message appeared on the screen alerting the responder to respond to the item to proceed to the next one. The study received ethical approval from the University of Verona and was conducted in accordance with the Declaration of Helsinki. The study findings are reported in accordance with the checklist for reporting results of Internet e-survey guidelines [28] and to strengthen the reporting of observational studies in epidemiology [29].

### Data analyses

Demographic characteristics and survey responses were examined with descriptive statistics, including frequencies and percentages. Frequencies and percentages of each response to predictors of the diagnosis, specialist consultations, treatment, management strategies, and satisfaction were analyzed according to years of post-specialization by means of the chi-squared test. Additional exploratory analyses have been conducted to compare the responses across the items of every single question (Supplementary Information). Statistical significance was set at *p* < 0.05. Bonferroni correction was applied where necessary. Analysis was performed using SPSS software (version 19).

## Results

Completed questionnaires were returned by 492 respondents (response rate, 16%; mean age ± SD, 49.11 ± 12.7; mean years of practice ± SD, 18.72 ± 13.37), with a fairly balanced male-to-female ratio. The majority were consultant neurologists (*n* = 470, 96%), while the remaining twenty-two were doctors in training (4%). Specialists and their subspecialty were: movement disorders (*n* = 116, 23%), cerebrovascular disease (*n* = 44, 9%), epilepsy (*n* = 53, 10%), other (general neurology: *n* = 77, 16%; headache: *n* = 20, 4%; neurodegenerative disease: *n* = 67, 14%; rehabilitation: *n* = 9, 2%; neurophysiology: *n* = 7, 1%; sleep *n* = 6, 1%; not specified *n* = 93, 19%) (Table 1).

**Table 1:** Sample demographics and years of practice

	Responses—no. (%)
Sex	
Male	241 (49)
Female	251 (51)
Age (years)	
< 40	152 (31)
41–50	104 (21)
51–60	122 (25)
> 60	114 (23)
Place of residence*	
Small city- population < 50,000	108 (22)
Middle-sized city—population > 50,000	260 (53)
Large city—population > 1 million	95 (19)
Years of practice (post-specialization)	
< 10	153
10–25	162
> 25	177

*Missed responses (*n* = 22, not specified)

### Practice with FND patients

Half of the sample (*n* = 247, 50%) thought that less than 10% of their patients presented symptoms without an organic cause. Many (41%, *n* = 203) reported a higher proportion of FND (10–25%); a few (7%, *n* = 34) stated that 25–50% of their patients might have a FND; very few (*n* = 3, 1%) reported that more than half of their patients were likely to have a FND or were unable to estimate how many of their patients might have a FND (*n* = 5, 1%).

### Terminology

A total of 1057 responses were collected, including free-text responses (*n* = 21). The majority of respondents (*n* = 303, 62%) chose more than one term. Overall, “Functional neurological disorders” (*n* = 374/1057, 35%) and “Somatization disorder” (*n* = 168/1057, 16%) were the two most frequent, followed by “Non-organic disorder” (*n* = 134/1057, 13%). The term “Functional neurological disorders” was selected 136 times on its own and 238 times together with psychology-related terms such as “Somatization disorder” (*n* = 117), “Psychogenic disorder” (*n* = 87), “Stress related disorder” (*n* = 21), “Conversion disorder” (*n* = 80), “Unspecific anxious syndrome” (*n* = 20), and “Depression” (*n* = 15). In addition, it was often selected with terms related to an undefined etiology (“Non organic neurological disorder”: *n* = 90; “Medically unexplained syndrome”: *n* = 5).

A field for free-text responses was included to capture any other terms; for example: psychogenic non epileptic seizures (*n* = 3), symptom description (*n* = 1), tremor under neurological assessment (*n* = 1), post-traumatic stress disorder (*n* = 1), neurological disease with negative neurological investigations (*n* = 1), gait disorders with negative neurological investigations (*n* = 1), type of disorder with negative neurological investigations (*n* = 1), functional neurological disorder in case of positive signs (*n* = 1), unspecific paresthesia (*n* = 1), supracortical disease (*n* = 1), dysfunctional disorder (*n* = 1), disease with unknown pathogenesis (*n* = 1), disease without an evident organic cause (*n* = 1).

### Probability that patients simulate symptoms

Many respondents believed that simulation was little (*n* = 328, 67%) or moderately probable (*n* = 131, 27%). Very few thought that simulation was highly probable (*n* = 9, 2%). At the two ends of the spectrum, no respondent stated that simulation was very highly probable and very few thought it highly unlikely that all patients feigned symptoms (*n* = 24, 5%).

### Explanation of symptoms

When asked about their preferred way to explain symptoms to their patients, the majority chose “Disorder due to abnormal functioning of the nervous system” (*n* = 284, 58%). Many chose “Psychogenic disorder” (*n* = 104, 21%) and few chose “Absent neurological disorder” (*n* = 57, 12%) or “Stress” (*n* = 31, 6%).

### Predictors of diagnosis

**Fig. 1:**
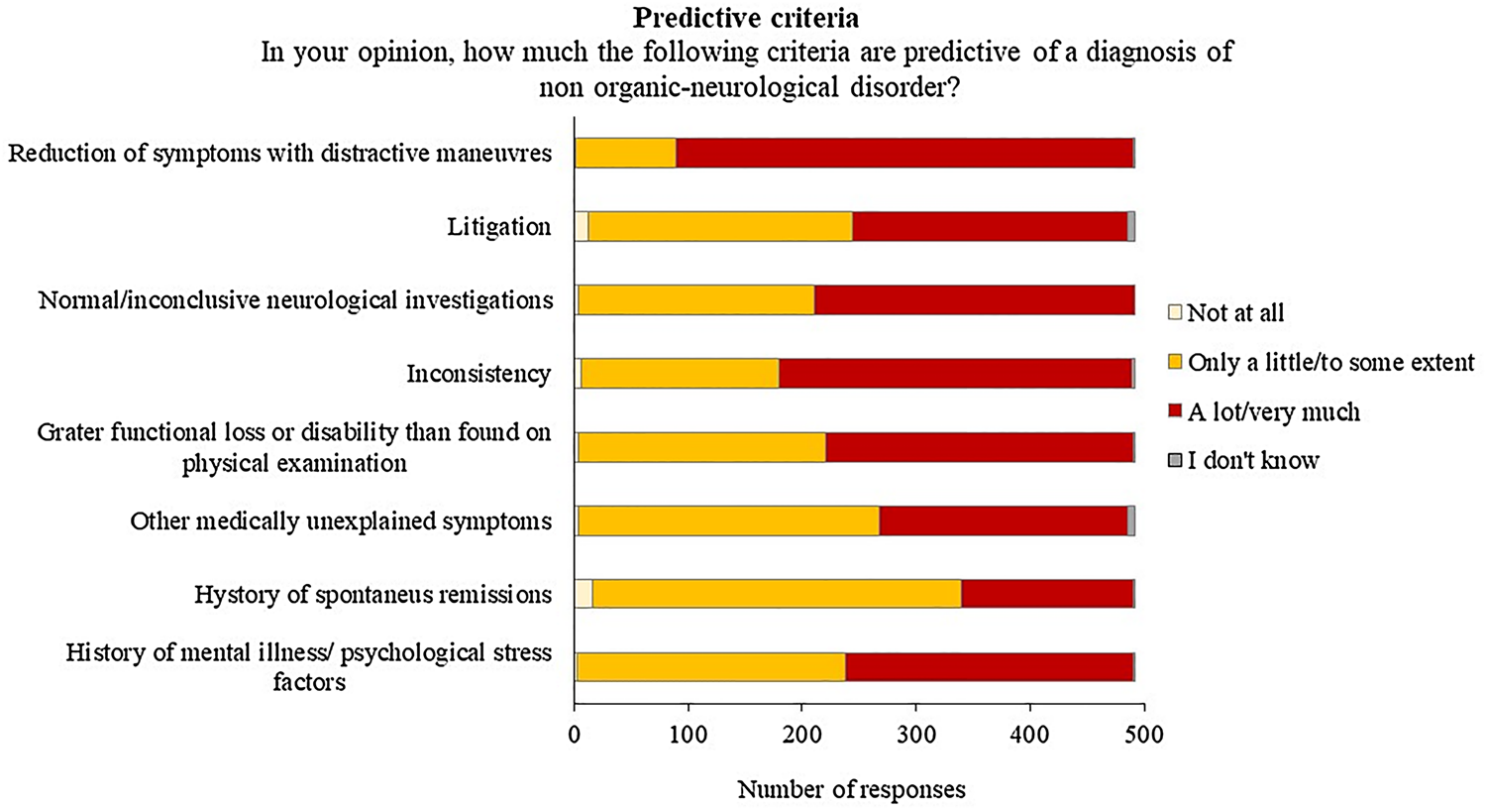
Distribution of responses for predictors of the diagnosis of FND

When asked to judge the extent to which certain diagnostic criteria were predictive for FND, the majority of respondents rated as “a lot” or “very much” predictive “Reduction in symptoms with distractive maneuvers” (*n* = 402, 82%), “Inconsistency” (*n* = 309, 62%), followed by “Normal or inconclusive neurological examination findings” (*n* = 281, 57%), “Greater loss of function or disability than found on physical examination” (*n* = 270, 55%), “Previous mental illness or psychological stress” (*n* = 253, 51%), and “Litigation” (*n* = 241, 49%). “Other medically unexplained symptoms” (*n* = 264, 54%) and “Spontaneous remissions” (*n* = 323, 66%) were mostly rated as “only a little” or “to some extent” predictive” (Fig. 1) (Supplementary Table 1). Predictors of diagnosis were not associated with years of post-specialization.

### Specialist consultation

**Fig. 2:**
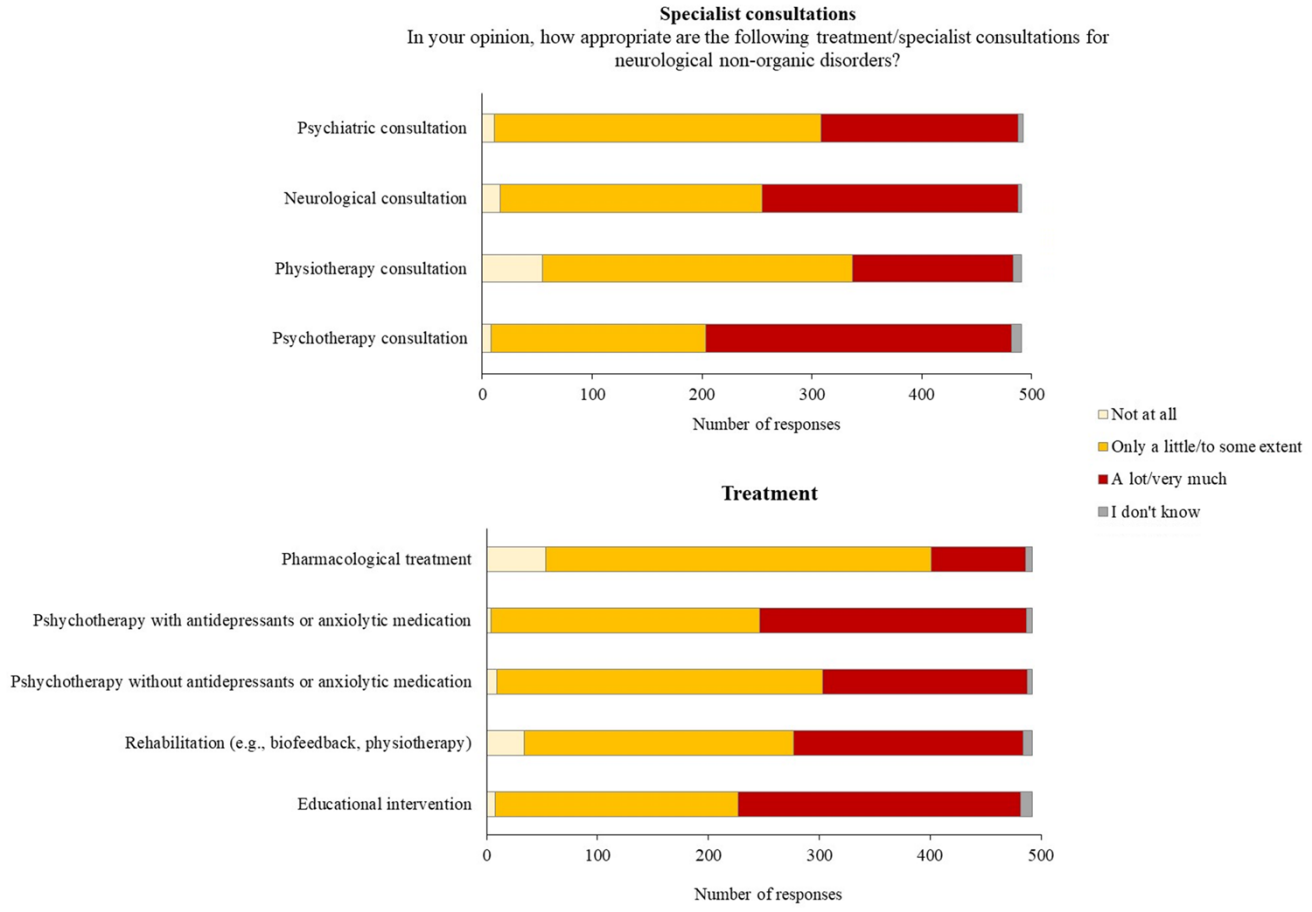
Distribution of responses for specialist consultation and treatment

“Psychotherapy consultation” was rated as “a lot” or “very much” adequate for FND by the majority of respondents (*n* = 279, 57%), with a higher proportion of neurologists with less than 10 years of practice (*χ*^2^ = 32.857, *p* < 0.001). “Neurological consultation” was frequently rated as “a lot” or “very much” adequate for FND (*n* = 233, 47%). “Psychiatric consultation” (*n* = 297, 60%) and “Physiotherapy consultation” (*n* = 282, 57%) were predominantly rated as “only a little” or “to some extent” adequate for FND (Fig. 2) (Supplementary Table 2). The degree of adequacy of “Physiotherapy consultation” was associated with more years of post-specialization (*χ*^2^ = 25.704, *p* < 0.001), meaning that the neurologists with more than 25 years of practice were more likely to believe “Physiotherapy consultation” as “only a little” or “to some extent” adequate for FND.

### Treatment

When asked to indicate the suitability of treatments for FND, “Educational interventions” (*n* = 255, 52%) and “Psychotherapy with antidepressant or anxiolytic medications” (*n* = 241, 49%) were the most frequently rated as “a lot” or “very much” adequate, while “Pharmacological treatment” (*n* = 348, 71%), “Psychotherapy without antidepressant or anxiolytic medications” (*n* = 294, 60%) and “Rehabilitation (e.g., biofeedback, physiotherapy)” (*n* = 243, 49%) were mostly rated as “only a little” or “to some extent” adequate (Fig. 2) (Supplementary Table 2). Years of specialization were associated with the responses given to “Educational interventions” (*χ*^2^ = 25.154, *p* < 0.001). More precisely, 39% (*n* = 99/255) of participants who rated “Educational interventions” as “a lot” or “very much” adequate had less than 10 years of post-specialization. Years of post-specialization also influenced responses to “Psychotherapy without antidepressant or anxiolytic medications” (*χ*^2^ = 30.328, *p* < 0.001), and “Rehabilitation (e.g., biofeedback, physiotherapy)” (*χ*^2^ = 36.345, *p* < 0.001). Longer years of post-specialization were associated with a higher proportion of “only a little” or “to some extent” responses to “Psychotherapy without antidepressant or anxiolytic medications” (*n* = 125/294, 43%) and to “Rehabilitation (e.g., biofeedback, physiotherapy) (*n* = 99/243, 41%)”.

### Management strategies

**Fig. 3:**
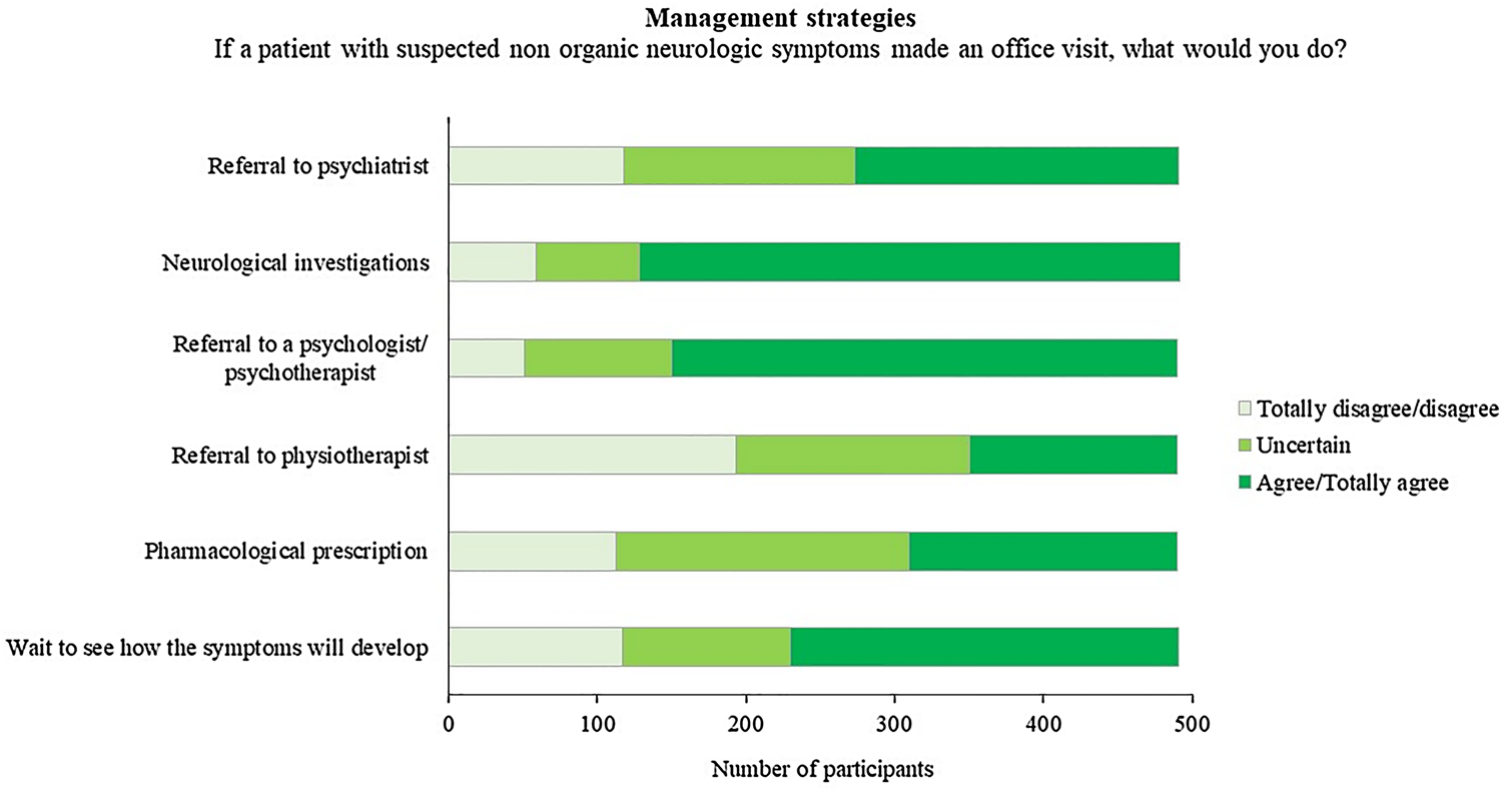
Distribution of responses for management strategies

The majority of respondents usually require “Neurological investigations” (e.g., fMRI) (“agree” or “extremely agree”: *n* = 364, 74%), “Refer patients to a psychologist or psychotherapist” (“agree” or “totally agree”: *n* = 340, 66%) or prefer to “Wait to see how symptoms develop” (“agree” or “totally agree”: *n* = 261, 53%). The management strategies more frequently excluded from clinical practice were “Referral to a psychiatrist” (“totally disagree” or “disagree”, n = 118, 24%), “Pharmacological prescription” (“totally disagree” or “disagree”: n = 113, 23%), and “Referral to a physiotherapist” (“totally disagree” or “disagree”: *n* = 193, 39%) (Fig. 3) (Supplementary Table 3).

“Referral to a psychologist or psychotherapist” (*χ*^2^ = 18.998, *p* = 0.001), “Wait to see how symptoms develop” (*χ*^2^ = 23.905, *p* = 0.001), “Pharmacological prescription” (*χ*^2^_4_ = 18.891, *p* = 0.001), and “Referral to a physiotherapist” (*χ*^2^ = 16.017, *p* = 0.003) were associated with years of post-specialization. The majority of respondents who usually refer patients to a psychologist or psychotherapist (*n* = 123/340, 36%) or prefer to wait to see how symptoms develop (*n* = 89/261, 34%) were neurologists with 10–25 years of post-specialization. Among those who mostly excluded “Pharmacological prescription” from their practice were respondents with less than 10 years of post-specialization (*n* = 42/113, 37%). Finally, respondents who mostly excluded “Referral to a physiotherapist” were neurologists with more than 25 years of post-specialization (*n* = 85/193, 44%).

### Satisfaction

When asked to rate their satisfaction in managing FND on an 11-point scale from 0 (not at all) to 10 (extremely satisfied), on average, respondents stated that they were satisfied with their care of patients with FND (4.90 ± 2.47). The majority (*n* = 290, 59%) rated their level of satisfaction between 5 and 10. Among these, the majority were neurologists with more than 25 years of post-specialization (*n* = 117/290, 40%) (*χ*^2^ = 7.903, *p* = 0.019).

### Role of neurologists

When asked about their clinical role in the management of FND*,* the majority (*n* = 273, 55%) gave more than one response, with “Following-up the treatment together with other specialists (psychiatrist, psychotherapist, physiotherapist)” the most frequent (*n* = 335, 68%), followed by “Make a diagnosis and recommend adequate treatment” (*n* = 291, 59%). Many chose “Referral to a specialist for the patient’s medical condition” (*n* = 99, 20%) and “Make a diagnosis and personally follow-up the patient” (*n* = 92, 19%)”, while very few believed their role was to provide “Educational intervention for patients and their families” (*n* = 16, 3%).

## Discussion

This web survey investigated opinions, knowledge and clinical experience with FND in a sample of Italian neurologists. The main findings suggest that a multidisciplinary approach to FND is emerging, although a full understanding of the disease in light of current advances still needs to be achieved.

Several studies from across the world investigated health professionals’ view on FND, many of them share similar results with our study [20, 24–26]. In our sample, the term “Functional neurological disorder” was most frequently selected. This is in line with a recent study showing that, in the last decade, the preferred term for this disorder has shifted to “functional” from “psychogenic” among neurologists with expertise in movement disorders [24]. Our study extends on these findings by demonstrating that this terminological transition occurred also among neurologists in different subspecialities. Patients and health professionals prefer this definition since it reduces the fear of social stigma, which is historically related to a psychiatric approach to this condition [30–33]. In line with this view, many respondents (58%) stated that they explain the disorder to their patients in terms of abnormal nervous system function rather than structural damage. This type of explanation is widely recommended, since it refers to the way in which symptoms manifest and may help to increase a patient’s understanding and acceptance of diagnosis [1]. However, a substantial percentage of respondents replied that they use definitions, like “Somatization disorder”, “Psychogenic disorder”, “Stress-related disorder”, and “Conversion disorder”. The use of “Conversion disorder” might reflect adherence to the DSM-5 classification, in which conversion disorder equates functional neurological symptoms as diagnostic labels. The use of the other psychological related terms, like “Somatization disorder”, “Psychogenic disorder” and “Stress-related disorder” might indicate, however, that a predominant psychological view of the disorder persists. Following this view, many (21%) said that they explain symptoms to their patients in terms of a psychogenic disorder, suggesting a psychological etiology of the disease. As a consequence, patients can feel misunderstood about their condition and reluctant to adhere to therapy [32]. Use of the term “non-organic disorder” was also relatively frequent; 10% of respondents stated that they usually explain the disorder as not caused by a neurological disease. Recent recommendations from the experts in the field discourage this approach since it conveys diagnostic uncertainty and the need to continue investigating for other causes [1, 30].

When asked about the probability that their patients simulate FND symptoms, few respondents stated that their patients do not feign symptoms, while many believed that patients produce symptoms deliberately. One possible explanation for respondents harboring suspicion about whether symptoms are feigned or not might be a lack of knowledge about FND pathophysiology. Recent behavioral and neuroimaging findings have provided evidence for an abnormal sense of agency in patients with FND [34, 35]. This high-level cognitive function distinguishes between voluntary and involuntary actions [36]. An altered sense of agency might explain the subjective loss of agency over abnormal movements (e.g., tremor) reported by patients with functional movement disorders. However, it does not explain other FND symptoms, where there are no abnormal movements (e.g., sensory loss, cognitive deficits). The fact that many respondents believe that patients might simulate their symptoms could also be due to a poor knowledge of diagnostic differences with factitious disorders and malingering. Promoting knowledge about these aspects is needed [6, 10]. What should also be taken into account that the suspicion that respondents harbor about symptom presentation might also be due to a lack of diagnostic tools for distinguishing illness deception from FND. In this regard, the study findings highlight the need for further research to provide useful tools for excluding deception in FND.

Our survey findings cast light on how Italian neurologists view the psychiatric and psychological aspects of FND, especially with regard to specialist consultation, treatment efficacy, and management strategies. For most of the past century, the psychiatric model predominated over a more comprehensive view of the disorder, involving neurobiological, psychological, and social factors [1]. The mechanism by which psychological stress is “converted” into physical symptoms [37] was long accepted for explaining the etiology of FND. Psychiatrists were considered appropriate health partners for FND patients. Over the last 20 years, however, the psychiatric view of FND has given way to a different view of the disorder, in which diagnosis is based on the identification of specific physical signs that allow to distinguish functional from organic disorders [1, 3, 8]. According to this, reducing symptoms with distractive maneuvers was rated among the most predictive criteria of FND, suggesting that many respondents take a diagnostic approach based on clinical examination rather than on the presence of psychological/psychiatric disturbances. Our study data show that the respondents considered psychotherapy and neurological consultation more appropriate than psychiatric consultation for FND. In line with a previous study [19], the present findings might suggest that a psychiatrist’s opinion is no longer relevant for the diagnosis of FND. By the same token, the observation that psychotherapy was considered more appropriate than psychiatric consultation might also suggest that psychological causes are important factors in managing patients with FND. Taking this approach, many respondents stated that they refer patients to a psychologist/psychotherapist and that they believed psychotherapy to be among the most appropriate treatments for FND. This is consistent with recent evidence suggesting that psychotherapy is effective in treating diverse forms of functional neurological disorders, including functional motor disorders [38] and psychogenic non-epileptic seizures [39]. Overall, it appears that a dualistic view of FND, in which brain and mind are separate components of human behavior, is being replaced by a view where both neurological and psychological aspects—brain and mind—are equally important in the diagnosis and treatment of such disorders [40, 41].

Neurologists need to understand the evidence on emerging therapeutic options for this complex disorder [8]. A multidisciplinary approach is now widely recommended, in which neurologists, psychotherapist, psychiatrists and physiotherapists are needed to improve patient care [8, 9, 15]. As in our previous study involving general practitioners [26], physiotherapy was rated lower than other management strategies. Physiotherapy remains underrecognized as a valuable approach to FND not only among general practitioners but also among neurologists. Recent evidence suggests that physiotherapy is useful for patients with functional motor disorders, which is one of the most common phenotypes of FND [42–44]. Moreover, psychiatric intervention needs to be taken into account. Recent studies suggest that psychological and psychiatric interventions should be regarded as pivotal, given the evidence that the beneficial effects of other treatments, like physiotherapy, might be lost in the long term [10]. Our findings indicate that a better understanding of how different therapeutic options work for FND is needed to improve care for patients with the condition. A purely pharmacological approach was considered to be of limited efficacy for treating FND and medication prescription was rarely the first intervention.

Another observation is that in their first intervention the respondents would order diagnostic or imaging studies to exclude neurological damage. This is in line with previous studies, in which neurological investigations (e.g., magnetic resonance imaging) were frequently requested for a diagnosis of FND [24–26]. Such investigations often produce negative results in FND patients and do not aid greatly in diagnosis. Differently, ad hoc neurological examination will usually catch positive clinical signs typical of FND [1]. Professional education in this area could improve confidence about diagnosis, thus reducing unnecessary ordering of additional investigations.

As reported in a more recent study involving neurologists with expertise in movement disorders [24], when asked about their role in patient management, the majority of respondents selected more than one option, suggesting that they take an active role in caring for their patients. According to recent recommendations on diagnosis [8], the majority of neurologists believe they have a multifaceted role: to establish a diagnosis, to suggest appropriate treatment, and to accompany their patients on the therapeutic pathway in collaboration with other health professionals (e.g., psychotherapists, physiotherapists, psychiatrists). The respondents stated that they preferred a multidisciplinary approach to the disorder and that they had a role in the diagnosis and management of the disorder.

Responses regarding patient education merit special comment. In line with a recent study [24], educational intervention was rated among the most appropriate treatments for FND. However, few respondents believed they had a role in patient education perhaps because they felt that other health professionals might be better trained for delivering education to patients and their families. In a multidisciplinary approach to FND all health professionals involved in the care of patients with FND (e.g., neurologist, psychologist, psychiatrist, physiotherapist) can advantageously deliver education to patients. For example, neurologists are encouraged to include an educational intervention in their neurological assessment; this will comprise an explanation of the positive nature of the diagnosis, advice about distraction techniques, and sources of information about the disorder [1]. Providing this information during neurological assessment could help patients understand the diagnosis and improve compliance with treatment [1]. Transferring knowledge and expertise in educational goals in neurological assessment could facilitate changes that would allow neurologists to improve efficacy in the management of FND. This might also improve satisfaction in managing FND patients, which was actually quite low in our sample. Like other health professionals (e.g., general practitioners, nurses, physiotherapists) [20–22, 24], many responders (41%) stated that they were dissatisfied with managing FND, which might be due to their outdated knowledge about the condition and may lie at the root of their difficulties in managing patients [19].

This study has several limitations. The survey response rate may not be fully representative of the entire neurologist community in Italy. It is also possible that only specialists interested in FND responded to the survey, thus limiting the generalizability of our findings. These limitations notwithstanding, with this exploratory study we provide novel insights into current opinions, knowledge, and clinical experience of a large cohort of Italian neurologists, working in different specialties. Investigating the actual view of neurologists on these disorders in a homogeneous geographical area, such as Italy, enabled us to catch local educational needs and develop an ad hoc educational program to improve care for these patients. The findings of the present study add knowledge about current practices in the management of FND in Italy. There is a need for guidance on the mechanisms underlying FND (e.g., suspicion about malingering is still present among Italian neurologists) and evidence-based treatment (e.g., multidisciplinary approach, including physiotherapy and psychiatric intervention). This should be provided to ensure that patients receive an appropriate explanation of their symptoms and management. The study enriches our knowledge of the current views of Italian health professionals about FND. The results of the present and the previous study [26] suggest that professional education is needed in our country to improve both primary and specialist care for FND. Specific educational courses for neurologists and for other health professionals (e.g., physiotherapists, psychiatrists, psychologists) could promote the dissemination of knowledge about FND, thus improving care for such patients.

## Supplementary Information

Below is the link to the electronic supplementary material.

Supplementary file1 (DOCX 36 KB)

## Data Availability

The raw data are available from the corresponding author upon request.
