# Supplementary material for: Opinion, knowledge, and clinical experience with functional neurological disorders among Italian neurologists: results from an online survey
Source: J Neurol. 2021 Oct 19;269(5):2549–59. doi: 10.1007/s00415-021-10840-y (PMC9021063; doi:10.1007/s00415-021-10840-y)
Supplement: Supplementary file 1 — Supplementary file1 (DOCX 36 KB) [file 415_2021_10840_MOESM1_ESM.docx]

**Supplementary Information**

**Survey Questions**

**1. Age _______**

**2. Sex**

☐ Male

☐ Female

**3. Year of practice (post-specialization) _______**

**4. Place of residence _______**

**5. Practice setting**

☐ Movement disorders

☐ Cerebrovascular disease

☐ Epilepsy

☐ Other (specify)

**6. In your practice, how many patients do you see in a week who might present neurological symptoms without an organic cause?**

☐ <10

☐ 10 - 25

☐ 25 - 50

☐ >50

☐ Don’t know

**7. Which of the following terms do you usually use to describe a clinical case characterized by neurological symptoms without an organic cause? [You can indicate more than one answer]**

☐ Functional neurological disorders

☐ Somatoform disorders

☐ Non-organic disorder

☐ Psychogenic disorder

☐ Conversion disorder

☐ Stress-related syndrome

☐ Depression

☐ Medically Unexplained Disorder

☐ Hysteria

**8. In your opinion, how probable is it that a patient with a non-organic neurological disorder simulates his/her symptoms?**

☐ Not at all

☐ Low probability

☐ Moderate probability

☐ High probability

☐ Very high probability

**9. Which of the following explanations would you use to describe non-organic neurological symptoms to your patient?**

☐ Disorder due to abnormal functioning of the nervous system

☐ Absent neurological disorder

☐ Psychogenic disorder

☐ Stress

☐ Other (specify)

**10. In your opinion, to what extent are the following criteria predictive of diagnosis of a non-organic neurological disorder?**

|  | Not at all | Only a little | To some extent | A lot | Very much | I don’t know |
| --- | --- | --- | --- | --- | --- | --- |
| Reduction in symptoms with distractive maneuvers | ☐ | ☐ | ☐ | ☐ | ☐ | ☐ |
| Litigation |  |  |  |  |  |  |
| Normal or inconclusive neurological exam findings | ☐ | ☐ | ☐ | ☐ | ☐ | ☐ |
| Inconsistency | ☐ | ☐ | ☐ | ☐ | ☐ | ☐ |
| Greater loss of function or disability than found on physical examination | ☐ | ☐ | ☐ | ☐ | ☐ | ☐ |
| Other medically unexplained symptoms | ☐ | ☐ | ☐ | ☐ | ☐ | ☐ |
| Spontaneous remissions | ☐ | ☐ | ☐ | ☐ | ☐ | ☐ |
| History of mental illness or psychological stress | ☐ | ☐ | ☐ | ☐ | ☐ | ☐ |

**11. In your opinion, how appropriate are the following treatment/specialist consultations for neurological non-organic disorders?**

|  | Not at all | Only a little | To some extent | A lot | Very much | I don’t know |
| --- | --- | --- | --- | --- | --- | --- |
| Pharmacological treatment of symptoms | ☐ | ☐ | ☐ | ☐ | ☐ | ☐ |
| Psychotherapy *with* antidepressants or anxiolytics | ☐ | ☐ | ☐ | ☐ | ☐ | ☐ |
| Psychotherapy *without* antidepressant or anxiolytics | ☐ | ☐ | ☐ | ☐ | ☐ | ☐ |
| Rehabilitation (e.g., biofeedback, physiotherapy) | ☐ | ☐ | ☐ | ☐ | ☐ | ☐ |
| Educational intervention | ☐ | ☐ | ☐ | ☐ | ☐ | ☐ |
| Psychiatric consultation | ☐ | ☐ | ☐ | ☐ | ☐ | ☐ |
| Neurological consultation | ☐ | ☐ | ☐ | ☐ | ☐ | ☐ |
| Physiotherapy consultation | ☐ | ☐ | ☐ | ☐ | ☐ | ☐ |
| Psychotherapy consultation | ☐ | ☐ | ☐ | ☐ | ☐ | ☐ |

**12. If a patient with suspected non-organic neurological symptoms came for an office visit, what would you do?**

|  | Totally disagree | Disagree | Uncertain | Agree | Totally agree |
| --- | --- | --- | --- | --- | --- |
| Referral to a psychiatrist | ☐ | ☐ | ☐ | ☐ | ☐ |
| Write an order for diagnostic tests | ☐ | ☐ | ☐ | ☐ | ☐ |
| Referral to a psychologist/psychotherapist | ☐ | ☐ | ☐ | ☐ | ☐ |
| Referral to a physiotherapist | ☐ | ☐ | ☐ | ☐ | ☐ |
| Write a drug prescription | ☐ | ☐ | ☐ | ☐ | ☐ |
| Wait to see how symptoms develop | ☐ | ☐ | ☐ | ☐ | ☐ |

**13. What is your level of satisfaction in managing a patient presenting with non-organic neurological symptoms?** [Select a number from 0 (no satisfaction) to 10 (high satisfaction)]

| No satisfaction |  | | | | | | | | | High satisfaction |
| --- | --- | --- | --- | --- | --- | --- | --- | --- | --- | --- |
| 0 | 1 | 2 | 3 | 4 | 5 | 6 | 7 | 8 | 9 | 10 |

**14. What is the neurologist’s role in the management of patients with non-organic neurological disorders? [You can indicate more than one answer]**

☐ Make a diagnosis and personally follow-up the patient

☐ Make a diagnosis and recommend appropriate treatment

☐ Refer the patient to a specialist for the condition

☐ Follow-up treatment together with other specialists (e.g., psychiatrist, physiotherapist, psychotherapist)

☐ Provide for education of the patient and family members

☐ Other (specify)

**Supplementary data analyses**

Responses to predictors of diagnosis, perceived usefulness of consultation, treatment adequacy for FND, and management strategies were further analyzed following a statistical approach based on rank distribution. With this different approach we wanted to explore potential differences across items with regard to the extent to which each item was predictive for a diagnosis of FND (question about predictors), the degree of adequacy of specialist consultations and treatment options (question about specialist consultations and treatment options), and the degree of agreement with each management strategy (question about management strategies). Before starting the data analysis, we converted the responses to each category into a 5-point Likert scale and assigned a score of 1 to the categories “not at all”/ “totally disagree”, 2 to the categories “only a little” / “disagree”, 3 to the categories “to some extent”/ “uncertain”, 4 to the categories “a lot”/ “agree”, and 5 to the categories “very much”/ “totally agree”. We then used the Wilcoxon signed rank test to compare responses across items, separately for each question. The “I don’t know” category was not included in these analyses, since it was not informative of the degree to which the corresponding item was predictive (i.e., predictors of diagnosis) or adequate (i.e., specialist consultation and treatment options for FND). Bonferroni correction was applied when necessary.

**Supplementary results**

*Predictors of diagnosis.* Respondents rated “Reduction in symptoms with distractive maneuvers” higher than any other item (all p<0.001). “Inconsistency” and “Normal or inconclusive neurological examination findings” were rated equally predictive of FND (p=0.037, critical p <0.002 after Bonferroni correction). These items were rated higher than all the others (p<0.001), except for “Greater loss of function or disability than found on physical examination”, which did not differ from “Normal or inconclusive neurological examination finings” (p =0.046, critical p <0.002 after Bonferroni correction). “Spontaneous remissions” was rated lowest among all options (all p< 0.001) (see Supplementary Table 1 for details).

*Specialist consultation.* When asked to judge the extent to which specialist consultations were appropriate for FND, “Psychotherapy consultation” was rated higher than the others (all p<0.001), followed by “Neurological consultation” and “Psychiatric consultation”. “Physiotherapy consultation” was rated the least appropriate for FND compared to all others (all p<0.001) (Supplementary Table 2).

*Treatment.* When asked to indicate the suitability of treatments for FND, on average, “Educational interventions” and “Psychotherapy with antidepressant or anxiolytic medications” were rated higher than “Rehabilitation (e.g., biofeedback, physiotherapy)” and “Psychotherapy without antidepressant or anxiolytic medications” (all p<0.001). “Pharmacological treatment” was rated lowest among all treatments (all p <0.001), indicating that a pharmacological approach was judged the least adequate for treating FND (Supplementary Table 2).

*Management strategies.* Responders were equally oriented towards: “Neurological investigations” and “Referral to a psychologist or psychotherapist” (p=0.68). These management strategies were rated higher than all others: “Wait to see how symptoms develop”, “Referral to a psychiatrist”, “Pharmacological prescription”, and “Referral to a physiotherapist” (all p<0.001). Scores for “Wait to see how symptoms develop” and “Referral to a psychiatrist” did not differ significantly (p=0.037, critical p <0.004 after Bonferroni correction) (Supplementary Table 3).

**Supplementary Tables**

**Supplementary Table 1.** Opinions about predictive criteria for FND diagnosis. Responses –no.(%)

|  | I don’t know | Not at all=1 | Only a little=2 | To some extent=3 | A lot=4 | Very much=5 | Average rating scores  (1 to 5) |
| --- | --- | --- | --- | --- | --- | --- | --- |
| Reduction in symptoms with distractive maneuvers | 1(0) | 1(0) | 15(3) | 73(15) | 198(40) | 204 (41) | 4.19 |
| Inconsistency | 3 (1) | 6(1) | 45 (9) | 129 (26) | 198 (40) | 111 (23) | 3.72 |
| Normal or inconclusive neurological exam findings | 0 (0) | 4 (1) | 50 (10) | 157 (32) | 194 (39) | 87 (18) | 3.63 |
| History of mental illness or psychological stress | 1 (0) | 3 (1) | 41 (8) | 194 (39) | 180 (37) | 73 (15) | 3.56 |
| Greater loss of function or disability than found on physical examination | 1 (0) | 4 (1) | 59 (12) | 158 (32) | 205 (42) | 65 (13) | 3.54 |
| Litigation | 7 (1) | 13 (3) | 75 (15) | 156 (32) | 160 (33) | 81 (16) | 3.41 |
| Other medically unexplained symptoms | 6 (1) | 4 (1) | 59 (12) | 205 (42) | 170 (35) | 48 (10) | 3.37 |
| Spontaneous remissions | 2 (0) | 16 (3) | 141 (29) | 182 (37) | 113 (23) | 38 (8) | 3.02 |
| Factors are listed in descending order of rating average. | | | | | | | |

**Supplementary Table 2.** Opinion about specialist consultation and treatment appropriateness for FND. Responses – no. (%)

|  | I don’t know | Not  at all=1 | Only  a little=2 | To some extent=3 | A lot=4 | Very much=5 | Average rating scores  (1 to 5) |
| --- | --- | --- | --- | --- | --- | --- | --- |
| **Specialist consultation** |  |  |  |  |  |  |  |
| Psychotherapy consultation | 9 (2) | 8 (2) | 50 (10) | 145 (30) | 189 (40) | 90 (18) | 3.56 |
| Neurological consultation | 3 (1) | 11(2) | 118 (24) | 179 (36) | 137 (28) | 43 (9) | 3.38 |
| Psychiatric consultation | 4 (1) | 11 (2) | 118 (24) | 179 (36) | 137 (28) | 43 (9) | 3.14 |
| Physiotherapy consultation | 8 (2) | 55 (11) | 141 (29) | 141 (29) | 105 (21) | 41 (8) | 2.82 |
| **Treatment** |  |  |  |  |  |  |  |
| Educational interventions | 10 (2) | 8 (2) | 58 (12) | 161 (33) | 182 (37) | 73 (15) | 3.46 |
| Psychotherapy with antidepressant or anxiolytic medications | 5 (1) | 4 (1) | 60 (12) | 182 (37) | 186 (38) | 55 (11) | 3.43 |
| Rehabilitation (e.g., biofeedback, physiotherapy) | 8 (2) | 34 (7) | 93 (19) | 150 (31) | 138 (28) | 69 (14) | 3.18 |
| Psychotherapy without antidepressant or anxiolytic medications | 4 (1) | 9 (2) | 119 (24) | 175 (36) | 141 (29) | 44 (9) | 3.16 |
| Pharmacological treatment | 6 (1) | 53 (11) | 187 (38) | 161 (33) | 76 (15) | 9 (2) | 2.56 |
| Factors are listed in descending order of average rating. | | | | | | | |

**Supplementary Table 3.** Management strategies. Responses - no. (%).

|  | Totally disagree=1 | Disagree=2 | Uncertain=3 | Agree=4 | Totally agree=5 | | Average rating scores  (1 to 5) |
| --- | --- | --- | --- | --- | --- | --- | --- |
| Neurological investigations | 10(2) | 49(10) | 69 (14) | 312 (63) | | 52 (11) | 3.71 |
| Referral to psychologist/  psychotherapist* | 10 (2) | 41 (8) | 99 (20) | 280 (60) | | 60 (12) | 3.68 |
| Wait to see how symptoms will develop^#^ | 24 (5) | 93 (19) | 113 (23) | 208 (42) | | 53 (11) | 3.35 |
| Referral to a psychiatrist^#^ | 30 (6) | 88 (18) | 155 (32) | 182 (37) | | 36 (7) | 3.21 |
| Pharmacological prescription* | 22 (5) | 91 (19) | 197 (40) | 173 (35) | | 7 (1) | 3.10 |
| Referral to a physiotherapist* | 62 (13) | 131 (27) | 157 (32) | 117 (24) | | 23 (5) | 2.81 |
| Factors are listed in descending order of rating average; * missed responses (n=2); ^#^missed responses (n=1) | | | | | | | |
